# Case Report: Diffuse cerebral lymphomatosis with superimposed multifocal primary CNS lymphoma

**DOI:** 10.3389/fradi.2024.1479282

**Published:** 2024-11-25

**Authors:** Elizabeth Huai-Feng Li, Claire Davila, Connor Zuraski, Jennifer Chang, Vanessa Goodwill, Nikdokht Farid

**Affiliations:** ^1^ School of Medicine, UC San Diego, San Diego, CA, United States; ^2^ Department of Neurology, UC San Diego, San Diego, CA, United States; ^3^ Department of Pathology, UC San Diego, San Diego, CA, United States; ^4^ Department of Radiology, UC San Diego, San Diego, CA, United States

**Keywords:** cerebral lymphomatosis, lymphomatosis cerebri, primary CNS lymphoma (PCNSL), diffuse leukoencephalopathy, diffuse tumorous infiltration, magnetic resonace imaging (MRI)

## Abstract

**Description:**

Cerebral lymphomatosis (CL) is a rare subtype of primary central nervous system lymphoma (PCNSL). In CL, atypical lymphoid cells diffusely infiltrate the cerebral parenchyma without forming a discrete mass as seen with PCNSL. We report a case of a 66-year-old woman with diffuse CL and superimposed areas of PCNSL. She presented with subacute cognitive decline and weakness. CSF studies showed lymphocytosis and IL-10 elevation. She became increasingly somnolent despite steroid and intravenous immunoglobulin trials, and she succumbed to the disease four months after symptom onset.

**Radiologic findings:**

Her initial non-contrast head CT showed ill-defined hypodensities in the periventricular and subcortical white matter, bilateral basal ganglia, and central pons, which corresponded to diffuse T2/FLAIR hyperintensities on brain MRI. No abnormal enhancement, diffusion restriction, or discrete mass was present initially. Subsequently, MR spectroscopy demonstrated abnormally elevated choline:creatine and decreased NAA peaks, suggesting a hypercellular process. One month later, MRI revealed increasingly confluent T2/FLAIR hyperintensities with new diffusion restriction in the right caudate and left hippocampus, as well as new hyperperfusion in the right caudate. Again, no mass or enhancement was identified in these areas. On autopsy, parenchymal pathology was mostly consistent with CL. However, there were two areas of frank PCNSL in the right caudate and left hippocampus, which corresponded to the new areas of abnormality on her last MRI despite lacking the typical radiologic features of PCNSL.

**Novel aspects:**

This is a unique case of CL with concurrent areas of PCNSL. Although CL is thought to be a distinct subtype of PCNSL, our case demonstrates that PCNSL may develop on a background of diffuse CL. In patients with subacute neurologic decline and MRI findings of diffuse leukoencephalopathy, diffuse CL should be considered.

## Introduction

Primary central nervous system lymphoma (PCNSL) is an uncommon type of primary brain malignancy, comprising only 6.6% of cases (1). Cerebral lymphomatosis (CL), also known as lymphomatosis cerebri, is a rare subtype of PCNSL and is defined as the diffuse infiltration of cerebral parenchyma by atypical lymphoid cells without the formation of a discrete mass. Histologically, 90% of PCNSLs are diffuse large B-cell lymphomas (DLBCL) (2), and the majority of reported cases of CL are also DLBCL (3, 4). Current treatment options for CL include methotrexate, corticosteroids, and radiation therapy (3). Patients receiving methotrexate therapy have a median survival of 13.8 months, which is significantly longer compared to other treatments. Administration of corticosteroids led to symptomatic improvement in some cases but has not been shown to improve overall survival (3, 5). Survival is significantly better in patients with less severe symptoms (3), suggesting that earlier diagnosis and treatment may prolong survival. However, CL remains difficult to diagnose due to its nonspecific clinical symptoms and imaging findings.

This report describes a patient who presented for generalized weakness and cognitive changes accompanied by diffuse leukoencephalopathy on imaging. Her nonspecific symptoms and imaging findings and her unresponsiveness to treatment presented a diagnostic challenge. After multiple hospital admissions, she was diagnosed with diffuse cerebral lymphomatosis with concurrent focal areas of primary CNS lymphoma.

## Case description

**Figure 1:**
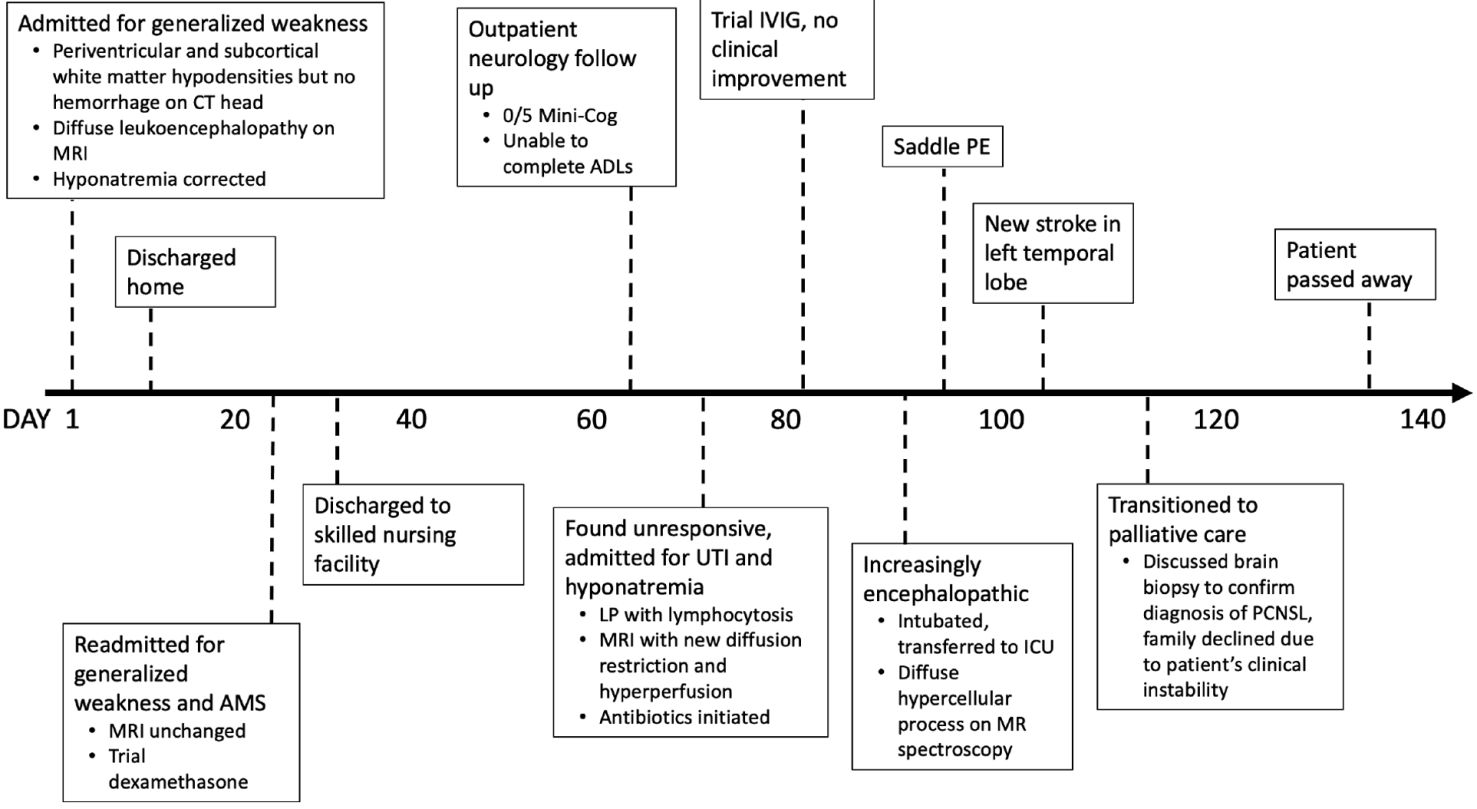
Clinical timeline. AMS, altered mental status; ADLs, activities of daily living; LP, lumbar puncture; IVIG, intravenous immunoglobulin; ICU, intensive care unit; PE, pulmonary embolism; PCNSL, primary CNS lymphoma.

A 66-year-old woman with an unremarkable past medical history presented with two weeks of decreased appetite, brain fog, and weakness resulting in multiple falls (Figure 1). Her neurologic exam was normal aside from mild bilateral lower extremity weakness and hyporeflexia. On admission, she was found to have severe hyponatremia and moderate leukocytosis but she was afebrile. Urine studies showed no evidence of urinary tract infection (UTI) and were consistent with syndrome of inappropriate antidiuretic hormone secretion (SIADH). Autoimmune labs, including ANA, ANCA, and SSA/B, were negative.

**Figure 2:**
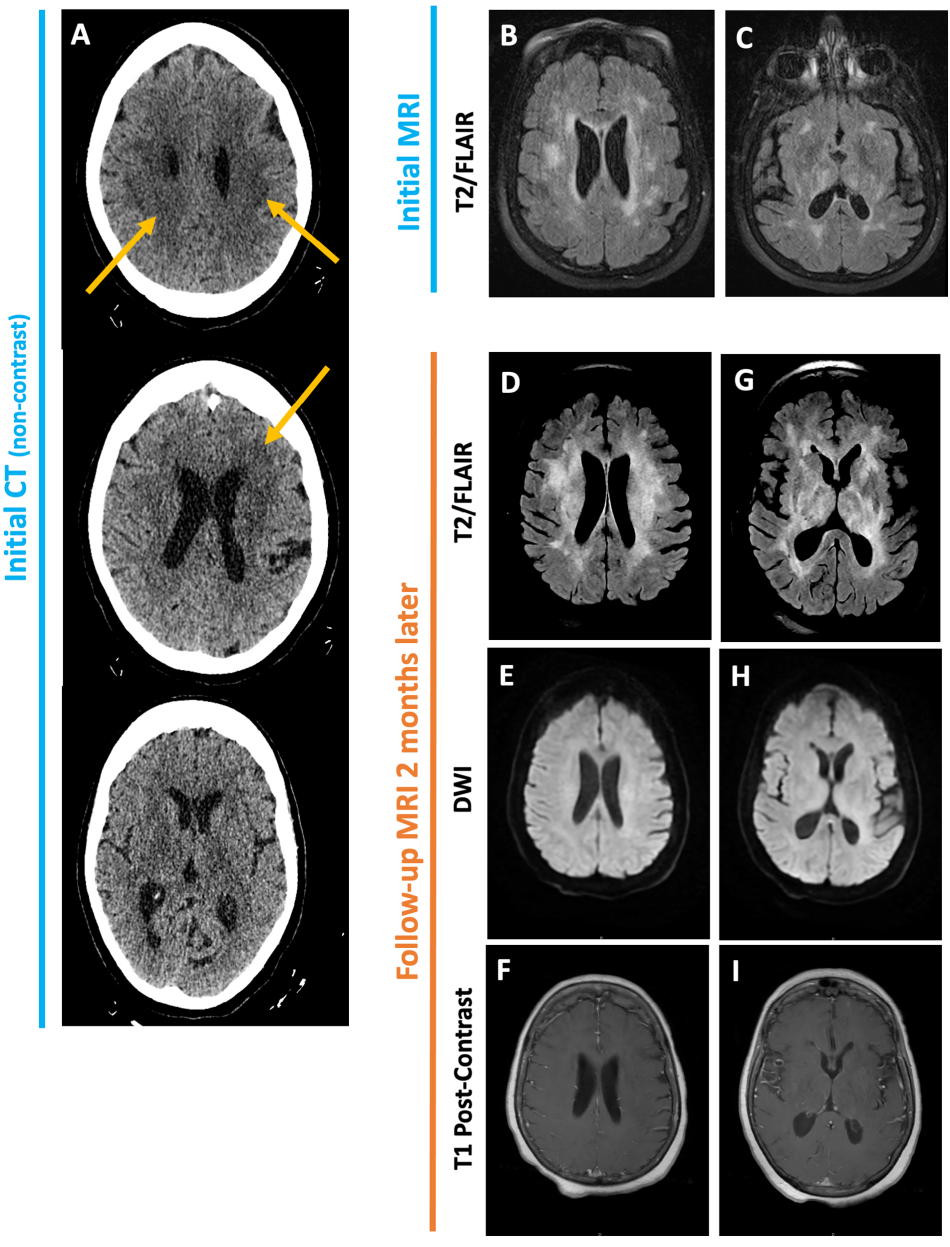
Initial non-contrast CT head **(A)** obtained for altered mental status shows ill-defined periventricular and subcortical white matter hypodensities. No evidence of intracranial hemorrhage, mass effect, or large territorial infarct. Initial MRI brain shows patchy T2/FLAIR hyperintensities in the periventricular and subcortical white matter as well as the bilateral basal ganglia **(B, C)**. Follow-up MRI two months later reveals increased confluence of these lesions **(D, G)**. No associated enhancement, diffusion restriction or mass effect is seen **(E, F, H, I)**.

Non-contrast head CT obtained on admission showed no intracranial hemorrhage, mass effect, or large territorial infarct but was notable for patchy hypodensities (Figure 2A). On MRI, these lesions involved the periventricular and subcortical white matter, bilateral thalami, and central pons and were T2/FLAIR hyperintense but did not enhance, restrict diffusion, or exhibit mass effect (Figures 2B,C). As her sodium was corrected at an appropriate rate with fluid restriction, salt tablets, and diuretics, her generalized weakness and confusion improved. Repeat MRI prior to discharge revealed no significant changes.

Of note, since her initial MRI was obtained prior to the correction of the hyponatremia, osmotic demyelination was not considered in the differential for her MRI findings. Rather, the non-specific MRI findings were attributed to chronic microvascular ischemic disease, and she was discharged home with outpatient neurology follow-up.

Ten days later, the patient re-presented to the emergency department for progressive generalized weakness and altered mental status. Neurologic exam noted poor attention, expressive aphasia, and slowed movements and speech. Labs revealed acute-on-chronic hyponatremia and mild leukocytosis. Given her worsening symptoms and persistent leukocytosis, the differential was expanded to include infectious, inflammatory, autoimmune, and toxic etiologies.

Cerebrospinal fluid (CSF) studies and additional serum labs were mostly unremarkable, however. C-reactive protein, angiotensin-converting enzyme, anti-thyroid antibodies, and antiphospholipid antibodies were within normal limits*.* A heavy metal panel testing for arsenic, lead, and mercury toxicity was also negative. A mild lymphocytosis (70% lymphocytes, 30% mononuclears) was seen on CSF cytology. MRI brain during this admission was unchanged compared to her prior MRI 12 days ago, and MRA showed no vascular abnormalities.

Despite a negative autoimmune workup, there was some concern for small vessel vasculitis due to her progressive encephalopathy in the setting of symmetric thalamic hyperintensities. A brain biopsy to evaluate the small vessels was recommended but declined, so an empiric course of dexamethasone with slow taper was started. Simultaneously, her hyponatremia was slowly corrected. The patient had minimal clinical improvement after starting steroids and was discharged to a skilled nursing facility. On discharge, she was alert and oriented to person, place, and time, but continued to have expressive aphasia.

At her neurology follow up one month later, she could no longer independently complete her activities of daily living and endorsed hallucinations and somnolence. She scored 0/5 on Mini-Cog and was unable to complete the MOCA. One week later, she was found to be febrile and unresponsive, so she was brought to the emergency department.

On presentation, she was febrile to 100.9 F and somnolent, oriented to person but not place, time, or situation, and had difficulty following commands. Neurology exam revealed bilateral upper greater than lower extremity weakness, hyporeflexic lower extremities, and intact sensation. Labs revealed acute-on-chronic hyponatremia and a UTI, so her acute change in mental status was attributed to infection. Lumbar puncture showed no evidence of CNS infection but was notable for persistent pleocytosis now with lymphocytosis (86% lymphocytes, 0% neutrophils).

**Figure 3:**
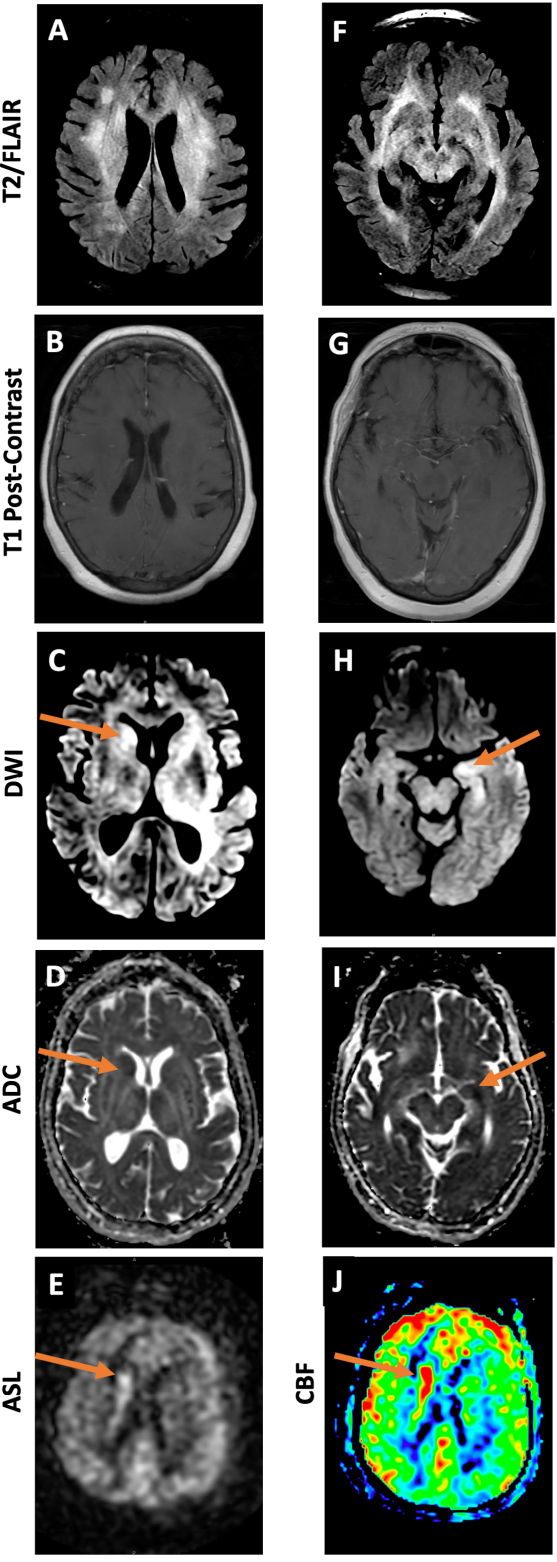
MRI brain 3 months after initial presentation demonstrates diffuse T2/FLAIR hyperintense lesions **(A, F)**. DWI and ADC images demonstrate restricted diffusion in the region of the right caudate **(C, D)** and left hippocampus **(H, I)**. Grayscale and color ASL perfusion images also demonstrate elevated perfusion in the region of the right caudate **(E, J)**. No abnormal enhancement or mass is seen in either area **(B, G)**.

On MRI, her periventricular and subcortical white-matter lesions appeared more confluent compared to prior (Figures 2D–I, 3A,F). Additionally, new asymmetric diffusion restriction was seen in the right caudate and left hippocampus and amygdala (Figures 3C–D,H–I). New asymmetric hyperperfusion was also noted in the right caudate (Figures 3E,J), however, these areas did not enhance on post-contrast imaging (Figures 3B,G). Metastatic disease was considered as a possible cause of chronic SIADH leading to hyponatremia, but no suspicious lesions were found on CT chest, abdomen, or pelvis. Electroencephalography showed mild diffuse abnormalities consistent with a generalized encephalopathy of unknown origin but without focal or epileptiform abnormalities.

Despite one week of broad-spectrum IV antibiotics and hyponatremia correction, the patient continued to have intermittent fevers and became increasingly somnolent. Additional labs, including paraneoplastic, autoimmune, metabolic, and infectious panels, revealed low serum IgG as well as mildly elevated IL6 and significantly elevated IL10 in the CSF, overall concerning for a primary CNS lymphoproliferative or autoimmune disorder. A course of IVIg was completed, but the patient did not clinically improve.

On MR spectroscopy, increased choline:creatine and decreased N-acetyl aspartate peaks were observed, indicating a hypercellular, likely neoplastic process. Cerebral angiography showed no evidence of vasculitis and bone marrow biopsy was negative for systemic lymphoma. Synthesizing all the available data, a primary CNS lymphoma was deemed the most likely diagnosis.

The patient continued to clinically decline and eventually required intubation. Shortly after, she was found to have a saddle pulmonary embolus and a new, small left temporal infarct. In discussion with the patient's family, the risks of attempting a brain biopsy to make a definitive diagnosis outweighed the benefits. She was transferred to palliative care and passed away three weeks later.

**Figure 4:**
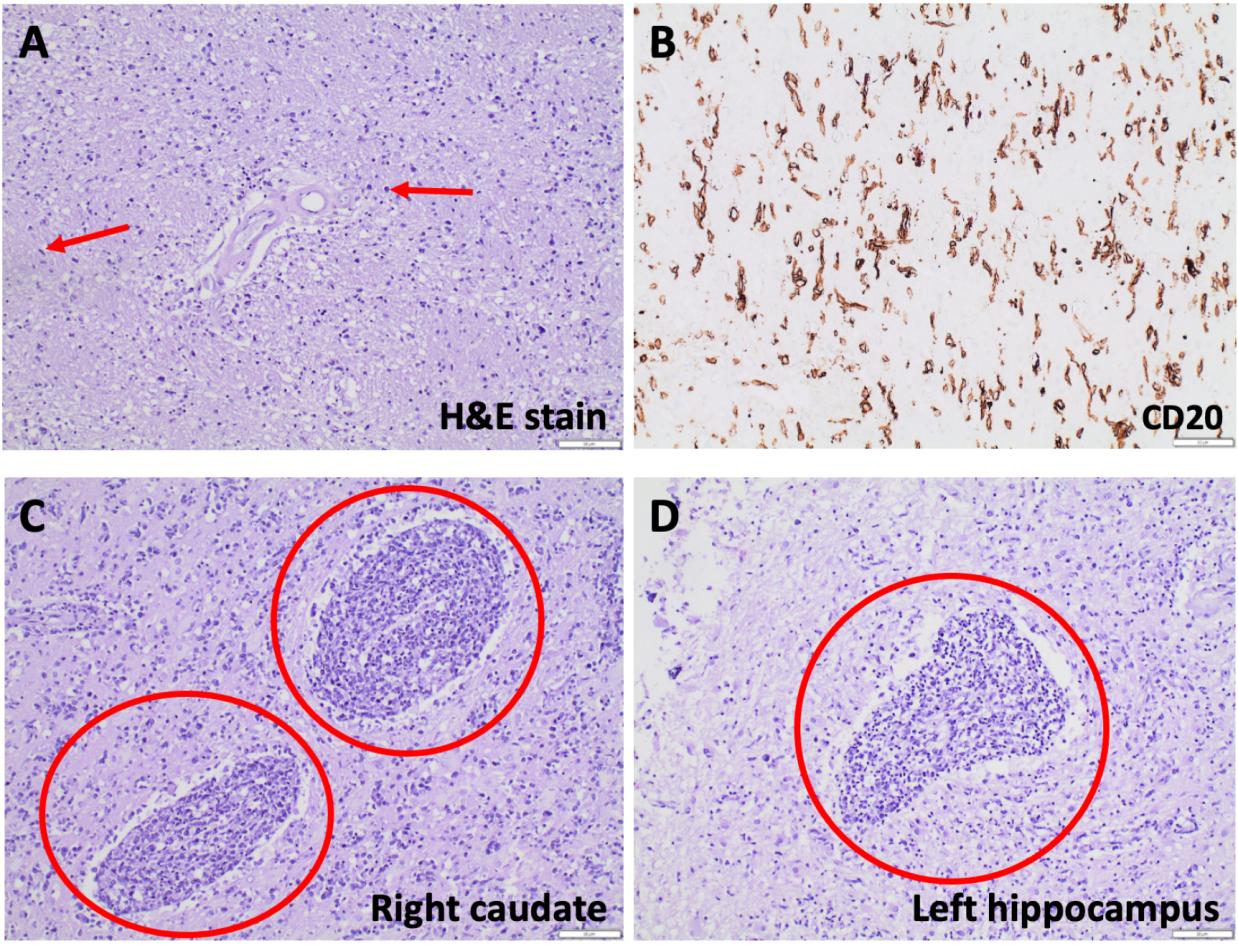
Hematoxylin-eosin (H&E) staining reveals parenchymal hypercellularity with diffuse infiltration of lymphocytic cells, some with large, round nuclei and others with gliomatosis cerebri-like elongate, angulated nuclei **(A)**. Lesional cells are CD20 positive on immunohistochemical staining **(B)**, many of which have Ki-67 positive nuclei. Archetypal features of primary CNS lymphoma, such as angiocentric, angioinvasive, discohesive malignant lymphocytes, are seen in the right caudate **(C)** and left hippocampus **(D)**.

On autopsy, gross examination of the brain showed mild global autolytic changes and a softened, friable right caudate nucleus. Microscopy revealed widespread parenchymal infiltration by atypical cells, which varied in morphology from elongate, angulate, and hyperchromatic nuclei to larger round to reniform nuclei with vesicular chromatin and prominent nuclei. CD20 positivity in the atypical cells confirmed B-cell lineage, and Ki-67 staining showed a high proliferative index. The diffuse pattern of involvement and staining characteristics were diagnostic of cerebral lymphomatosis. In addition, two more discrete foci of angioinvasive, discohesive, malignant lymphocytes seen in the right caudate and the left hippocampus had archetypal features of primary CNS lymphoma (Figure 4).

## Discussion

We report the first case of cerebral lymphomatosis (CL) with superimposed multifocal areas of primary CNS lymphoma (PCNSL). Our patient presented with subacute neurologic symptoms that were initially attributed to hyponatremia secondary to SIADH. Initial imaging revealed diffuse, non-enhancing, bilateral T2/FLAIR-hyperintensities. Although her symptoms resolved with correction of hyponatremia at first, her mental status progressively worsened over the following three months. Infectious and autoimmune workup was consistently negative, and she did not respond to empiric trials of high-dose dexamethasone or IV immunoglobulin. Notably, repeat MRI three months after her initial presentation revealed new areas of asymmetric diffusion restriction and hyperperfusion that directly corresponded to pathologically proven foci of PCNSL.

White matter changes can be seen in numerous disease processes. Changes that remain relatively stable over time are commonly due to chronic microangiopathy. Chronic demyelinating disease may also be considered if the lesions have a more characteristic periventricular distribution with radial orientation of the lesions and additional involvement of the corpus callosum and possibly the infratentorial white matter. However, in cases with rapidly progressive white matter changes, acute/subacute processes should be considered including acute disseminated encephalomyelitis, acute demyelinating disease such as neuromyelitis optic (NMO) spectrum disorder and myelin oligodendrocyte glycoprotein antibody-associated disease (MOGAD), infectious etiologies, toxic/metabolic etiologies, autoimmune and paraneoplastic etiologies, posterior reversible encephalopathy syndrome, vasculitis, and adult-onset leukodystrophy. CSF analysis is critical to differentiate these disease processes and, in some cases, additional workup such as digital subtraction angiography or even brain biopsy may be indicated.

In our case, a broad differential diagnosis was considered for the patient's generalized weakness and worsening encephalopathy. A metabolic process was initially considered, given the patient's hyponatremia. However, when her symptoms gradually worsened despite sodium correction, the differential was expanded. Workup for autoimmune etiologies—including systemic lupus erythematosus, Sjogren's syndrome, sarcoidosis, acute disseminated encephalomyelitis, HTLV-1 associated diseases, antiphospholipid syndrome, and thyroid diseases—was negative. Testing for infectious causes, including viral, bacterial, tuberculosis, fungal, and parasitic were all negative. Malignancy screening with CT chest, abdomen, and pelvis as well as CSF paraneoplastic panels were unremarkable. No environmental causes (e.g., heavy metal toxicity) were identified. Elevated levels of IL10 in the CSF pointed toward a CNS lymphoproliferative disorder and MR spectroscopy confirmed a hypercellular process. As the patient and family declined brain biopsy by this point, a presumptive diagnosis of CNS lymphoma was made and autopsy confirmed CL in addition to focal areas of PCNSL.

This case shares some similarities with previously reported cases of CL and furthers the limited pool of literature characterizing the typical presentation of such a rare condition (6, 7). A review of 45 case reports found that cognitive changes, gait ataxia, and motor deficits were the most common presenting symptoms (8). CSF studies were abnormal in the majority of cases, most commonly due to increased cell counts, but neoplastic lymphocytes were not present in any of the CSF cytology reports. Therefore, CSF studies lacking neoplastic lymphocytes cannot reliably rule out CL. However, a few studies have also suggested that elevated IL-10 levels may be a possible marker of CL (9–12), and our case supports this theory.

One notable abnormality in our case was the patient's persistent hyponatremia. Common causes of SIADH include medication side effects, paraneoplastic syndromes, persistent limbic system activation (e.g., secondary to pain), and CNS disorders such as stroke, infection, or psychosis. In retrospect, given that review of the patient's home medications and malignancy workup were unremarkable, her hyponatremia was possibly due to infiltration of the hypothalamus by CL, resulting in SIADH. Prior studies have shown that invasion of the hypothalamus by sarcoidosis can cause SIADH (13). Moreover, Oishi et al. 2023 previously postulated that SIADH may be a presenting sign of PCNSLs that involve the hypothalamus, but to our knowledge, no studies to date have reported this association specifically with diffuse cerebral lymphomatosis (14, 15). This case highlights the importance of elucidating the underlying cause of SIADH. In patients with persistent SIADH and evidence of diffuse abnormality on brain imaging who have an otherwise negative workup, neoplastic processes such as CL should be included on the differential.

In terms of imaging, CL remains difficult to diagnose because its associated findings are also seen in many other conditions, including gliomatosis cerebri, infection, inflammatory conditions, and toxic/metabolic disorders (9, 16, 17). If a neoplastic process is suspected, a lower threshold to perform MR spectroscopy sooner in the workup could reveal a hypercellular process and lead to earlier diagnosis and treatment. Moreover, PCNSL and CL typically have distinct features on MRI. While PCNSL presents with single or multiple masses that enhance and may exhibit mass effect, CL presents with diffuse T2 hyperintensities affecting both gray and white matter but without mass effect. CL lesions usually do not enhance initially but may enhance later with disease progression. This is thought to be due to the eventual breakdown of the blood brain barrier by worsening disease (8). In our case, no contrast enhancement or mass effect was seen on any of the patient's serial MRIs despite the rapid progression and increased confluence of her lesions.

Additionally, it has been suggested that sparing of the caudate head and putamen on MRI may be unique to CL and could clue physicians in to its diagnosis (18). To our knowledge, only one case of caudate nuclei involvement has been reported and was found to be a T-type CL (19). In our case, no caudate involvement was initially observed, but repeat imaging three months later revealed new diffusion restriction and hyperperfusion of the right caudate as well as diffusion restriction in the left hippocampus. The pathology of the parenchymal white matter supports a diagnosis of diffuse CL, but the pathology of the right caudate and left hippocampus exhibits classic features of PCNSL. Therefore, this case is particularly unique because (1) the multifocal areas of pathology-proven PCNSL did not enhance on imaging, and (2) these foci of PCNSL developed on a background of diffuse CL, which suggests a possible progression or evolution from CL to PCNSL. It remains unknown if the foci of PCNSL in this case would have eventually demonstrated contrast enhancement and/or mass effect with continued disease progression.

The current standard of treatment of PCNSL involves methotrexate, which this patient did not receive. Although she was treated with empiric courses of steroids and IVIg, no significant clinical or imaging improvement was seen. Thus, our case also highlights the chronological characterization of the natural history of rapidly progressive CL.

Ultimately, cerebral lymphomatosis remains difficult to diagnose due to its nonspecific clinical and neuroimaging features. Extensive workup to rule out other infectious, inflammatory, autoimmune, and toxic/metabolic etiologies is often required, and brain biopsy to confirm the diagnosis may not be feasible in clinically unstable patients. Moreover, temporary clinical improvement with initial symptomatic management or steroid treatment may further contribute to delays in diagnosis. The median time from initial presentation to diagnosis for all types of PCNSLs in immunocompetent patients is around 40 days (20, 21). However, a survey of 25 CL cases found that the median time from symptom onset to first consultation is 2 months and to definitive diagnosis is 4.5 months (3).

## Conclusion

This report features a unique case of diffuse cerebral lymphomatosis with concurrent multifocal areas of primary CNS lymphoma. Several diagnostic challenges – including confounding symptoms, variable and nonspecific neuroimaging findings, and diffuse lesions that may be difficult to target on biopsy – may inadvertently lead to delays in diagnosis. Since better outcomes are seen with earlier diagnosis and treatment, physicians should have high clinical suspicion for cerebral lymphomatosis in elderly patients who present with subacute, rapidly progressive cognitive decline and diffuse leukoencephalopathy on neuroimaging.

## Data Availability

The original contributions presented in the study are included in the article/Supplementary Material, further inquiries can be directed to the corresponding author.
